# A Method for Parameter Identification of Composite Beam Piezoelectric Energy Harvester

**DOI:** 10.3390/s21217213

**Published:** 2021-10-29

**Authors:** Xuhui Zhang, Chao Zhang, Lin Wang, Luyang Chen, Xiaoyu Chen, Dongmei Xu, Hongwei Fan, Fulin Zhu

**Affiliations:** 1 College of Mechanical Engineering, Xi’an University of Science and Technology, Xi’an 710054, China; 17205201064@stu.xust.edu.cn (C.Z.); 18205018017@stu.xust.edu.cn (L.W.); chenluyang@stu.xust.edu.cn (L.C.); 19105016004@stu.xust.edu.cn (X.C.); dongmeixu@xust.edu.cn (D.X.); hw_fan@xust.edu.cn (H.F.); 20205224060@stu.xust.edu.cn (F.Z.); 2 Shaanxi Key Laboratory of Mine Electromechanical Equipment Intelligent Monitoring, Xi’an 710054, China

**Keywords:** piezoelectric energy harvester, stiffness, damping, parameter identification

## Abstract

This paper proposes a parameter identification method for the multiparameter identification study of the linear–arch composite beam piezoelectric energy harvester. According to the voltage response characteristics of the system under short-circuit conditions, the mechanical equation is solved by transient excitation, combined with the backbone curve theory and logarithmic attenuation method, to obtain the system’s linear damping, linear stiffness, and nonlinear stiffness. According to the voltage response characteristics of the system under open-circuit conditions, combined with the electrical equations, the system electromechanical coupling coefficient and equivalent capacitance coefficient are obtained; numerical simulation results show that the identification parameters have good accuracy. Finally, an experimental platform was built for verification, and the results show that the method has high accuracy and practicability.

## 1. Introduction

With the rapid development of wireless sensor network technology, low-power wireless sensors have been widely used in large-scale equipment state detection [1]. Vibration energy harvesting technology has attracted much attention due to its application prospects in microelectronic equipment power supply and equipment condition monitoring [2,3,4,5,6,7]. The use of piezoelectric self-capture energy-powered wireless monitoring technology to replace traditional wired monitoring is expected to greatly alleviate the wiring difficulties and power supply difficulties of online monitoring of complex environment equipment in coal mines [8]. For this reason, some experts and scholars have proposed a variety of structural energy harvesters and performed dynamic modeling and response characteristics analysis [9,10,11,12,13]. However, it is difficult to accurately express the mechanical and electrical parameters in the model through traditional mathematical modeling methods, which results in certain errors in the system dynamic response analysis and experimental results. Therefore, a lot of attention has been paid to the parameter identification technology of piezoelectric energy harvesting systems. Feldman et al. [14] proposed two accurate identification methods; the stiffness and damping parameters of the nonlinear vibration system are obtained based on the free vibration of the classical nonlinear system, and the feasibility of the method is verified numerically.

Lu et al. [15] proposed steady-state excitation parameter identification and transient excitation parameter identification methods for a vibration system containing both nonlinear stiffness and nonlinear damping. Taking the vibration isolation system with nonlinear stiffness and nonlinear damping as an example, the two types of parameter identification methods given are verified by numerical simulation. The results show that the results of the two identification methods are relatively consistent, and the steady-state parameter identification method is more accurate and convenient than the two, but an experimental platform has not been built to verify the identification algorithm. Yuan et al. [16,17,18] used the restoring force surface method and the Hilbert transform method to study the nonlinear parameter identification of piezoelectric twin-crystal thin plates. According to the experimental results, the accuracy of the displacement–stiffness function is proved and the nonlinear characteristics of the system are accurately displayed. Zhou [19] proposed a tri-stable piezoelectric energy harvester and identified the damping and electromechanical coupling coefficients of the system through the genetic algorithm (GA) method. The results show that the algorithm is very accurate in identifying parameters. Chen et al. [20] proposed an electrical parameter identification method for the laminated disc piezoelectric energy harvester to identify the electromechanical coupling coefficient and equivalent capacitance coefficient of the system. The result is highly consistent with the theoretical value and verifies the accuracy of the algorithm.

Alper Erturk et al. [21] used a nonlinear least-squares optimization algorithm for a cantilever beam piezoelectric energy harvester to identify the electromechanical coupling coefficient and damping coefficient of the system. The comparison of theoretical and experimental results proved the effectiveness of the algorithm, and it was found that adding coupling nonlinearity will shift the nonlinear response of the system to the right. Adnan Kefal et al. [22] proposed a new parameter identification algorithm based on Lyapunov’s theory, and by analyzing the time-domain response of the array piezoelectric bimorph under the random vibration of the low-frequency structure, the array parameter values were identified, and the accuracy of the algorithm was proved. Porfiri et al. [23] proposed two techniques for estimating the electromechanical coupling coefficient and piezoelectric modal capacitance; the effectiveness of the algorithm was proved by the comparison of bi-crystal piezoelectric cantilever beam experiment and finite element simulation analysis. Delpero et al. [24] proposed a method of measuring the electromechanical coupling coefficient and applied it to experiments on piezoelectric energy harvesters of different sizes; the experimental results are in good agreement with the analysis and prediction. Binh Duc Truong et al. [25] proposed a parameter identification method based on least-squares minimization, which estimates the damping and stiffness coefficients of the system according to the frequency response function of the system dynamics equation, and the algorithm is suitable for linear and nonlinear systems.

**Figure 1:**
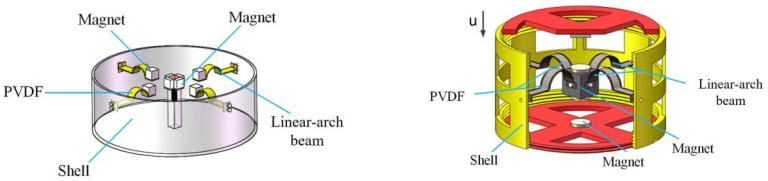
Multidirectional piezoelectric energy harvester based on linear–arch composite beam.

Excitation in the environment is usually low-frequency and multidirectional; traditional linear beams can only respond to excitation in a single direction, which is difficult to achieve in practical applications. In order to realize the self-powering of wireless monitoring nodes in coal mines. Zhang [26] designed two linear–arch composite beam multidirectional piezoelectric energy harvesters, as shown in Figure 1; the energy harvester can collect multidirectional vibration energy. The introduction of the arch structure makes the system dynamics model complicated, and it is difficult to achieve accurate modeling with the traditional modeling method. Therefore, this paper proposes a method to identify the system mechanical and electrical parameters of the linear–arch composite beam piezoelectric energy harvester. According to the voltage response characteristics of the system under short-circuit conditions, the transient excitation of the mechanical equations is solved. Combining the ridge theory and logarithmic attenuation method, the system’s linear damping, linear stiffness, nonlinear stiffness, and other mechanical parameters are obtained with an identification procedure, according to the voltage response characteristics of the system under open-circuit conditions; combined with the electrical equations, electrical parameters such as the electromechanical coupling coefficient and equivalent capacitance coefficient of the system are obtained. The identified parameters are finally compared with the experimental results to verify the correctness of the procedure. The parameter identification method proposed in this paper is of great significance for guiding the modeling under complex nonlinear conditions. It is helpful in improving the accuracy of theoretical modeling and providing theoretical guidance for optimizing system parameters.

## 2. The Structure and Theoretical Model of the Piezoelectric Energy Harvester

**Figure 2:**
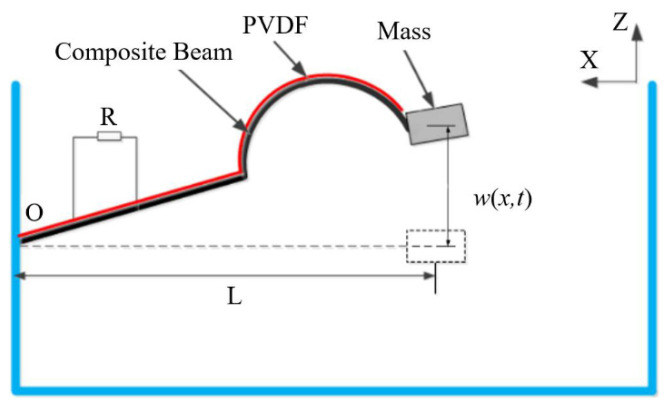
Schematic diagram of the structure of the PEH-C.

The piezoelectric vibration energy harvester based on a composite beam (PEH-C) is shown in Figure 2. The system is mainly composed of a base, a load resistor, a composite beam, a piezoelectric film, and a mass. In order to enhance the directional sensitivity of the system to vibration sources in the real environment, the composite beam is made of a combination of linear and arch. The horizontal length of the composite beam is L. Under the excitation of external vibration along the *Z*-axis, the composite beam vibrates up and down, and the piezoelectric material pasted on the beam deforms at the same time. In this way, the positive piezoelectric effect of the piezoelectric material is used to convert the vibration energy in the actual environment into electrical energy.

w(x,t) is the displacement of a point on the linear–arch composite beam along the *Z*-axis at time t, According to the Rayleigh–Ritz theory, the relative displacement w(x,t) of linear–arch composite beam vibration can be discretized as a combination of modes

(1) $$w\left(x,t\right)=\overset{n}{\underset{i=1}{\sum }}\varphi_{i}\left(x\right)x\left(t\right)$$

where $\varphi_{i}\left(x\right)$ represents the mode shape of the ith mode of the linear–arch composite beam and x(t) represents the generalized modal coordinates.

For the linear–arch composite beam in this paper, one end is clamped and fixed on the base, and the other end is free. The mode of the linear–arch composite beam is difficult to obtain; therefore, the allowable function is adopted to simplify it. The allowable function can be expressed as [27]

(2) $$\varphi_{i}\left(x\right)=1-\cos\left[\frac{\left(2i-1\right)\pi x}{2L}\right]$$

Since the excitation of piezoelectric energy harvester is mainly low-frequency, the first-order modal bending vibration of the linear–arch composite beam plays a leading role. This paper only considers the first-order mode of the linear–arch composite beam; therefore, i=1, $\varphi_{1}\left(L\right)=1$, and w(L,t) = x(t).

For the piezoelectric vibration energy harvester based on composite beam (PEH-C) system, considering the structural nonlinearity, the generalized Hamilton principle and Lagrange function are used to establish the system parameter model, and the dynamic equation of the PEH-C system is obtained [28]:

(3) $$\begin{array}{c} m\ddot{x}\left(t\right)+c\dot{x}\left(t\right)+\beta x{\left(t\right)}^{3}+\alpha x\left(t\right)-\vartheta v\left(t\right)=-H_{s}\ddot{z}\left(t\right)\\ \vartheta \dot{r_{1}}\left(t\right)+c_{p}\dot{v}\left(t\right)+v\left(t\right)/R=0 \end{array}$$

where m is the composite beam mass, c is the composite beam first-order damping, α is the linear stiffness coefficient, β is the nonlinear stiffness coefficient, ϑ is the electromechanical coupling coefficient, x(t) is the response displacement,$\;H_{s}$ is the system foundation excitation coefficient, v(t) is the output voltage, z(t) is the external basic incentive, $c_{p}$ is the system equivalent capacitance coefficient, and R is the resistance value of external load.

## 3. Theoretical Modeling of Parameter Identification Method

From Equation (3), when the system is in a short-circuit condition, the output voltage of the system is 0, so the mechanical equation in the system dynamics equation can be rewritten in the following form:

(4) $$m\ddot{x}\left(t\right)+c\dot{x}\left(t\right)+\alpha x\left(t\right)+\beta x^{3}\left(t\right)=f\left(t\right)$$

where f(t) is the external excitation. x(t) is the end displacement, and the positive direction of the *Z*-axis shown in Figure 2 is the positive direction of the displacement.

We select external excitation as unit pulse excitation; that is, f(t)=δ(t). Then, Equation (4) can be written as

(5) $$m\ddot{x}\left(t\right)+c\dot{x}\left(t\right)+\alpha x\left(t\right)+\beta x^{3}\left(t\right)=0$$

Then the solution of Equation (5) can be set as

(6) x(t)=A(t)cos[φ(t)]

In the formula, A(t) is the amplitude of the system over time, and φ(t) is the phase angle. When the system moves along the main vibration curve under the condition of free vibration, the instantaneous frequency and instantaneous phase angle have the following relationship:

(7) $$\varphi \left(t\right)=\int_{0}^{\tau }\omega_{d}\left(\tau \right)d\tau$$

In the formula, $\omega_{d}$ is the instantaneous frequency.

For the system described by the Equation (5), the instantaneous frequency analytic formula that changes with time is [29]

(8) $$\omega^{2}{}_{d}\left(t\right)=\omega^{2}{}_{dd}\left(t\right)+\frac{3}{4}\frac{\beta }{m}A^{2}\left(t\right)$$

In the formula, the damped natural angular frequency $\omega_{dd}=\omega_{n}\sqrt{1-\zeta^{2}}$, the undamped natural angular frequency $\omega_{n}=\sqrt{\alpha /m}$, and damping ratio $\zeta =c/\left(2\sqrt{\alpha \times m}\right)$; we assume $A\left(t\right)=A_{0}e^{-\zeta \omega_{n}t}$ according to the theory of multiscale method and combine Equations (7) and (8) to obtain

(9) $$\varphi \left(t\right)\approx \sqrt{1-\zeta^{2}}\omega_{n}t+\frac{3\beta }{16\alpha \sqrt{1-\zeta^{2}}}A_{0}{}^{2}\left(1-e^{-2\zeta \omega_{n}t}\right)$$

(10) $$\omega_{d}\approx \sqrt{1-\zeta^{2}}\omega_{n}+\frac{3\beta \zeta \omega_{n}}{8\alpha \sqrt{1-\zeta^{2}}}A_{0}{}^{2}e^{-2\zeta \omega_{n}t}\;$$

Substituting Equation (9) into (6), we can obtain

(11) $$x\left(t\right)\approx A_{0}e^{-\zeta \omega_{n}t}\cos\left[\sqrt{1-\zeta^{2}}\omega_{n}t+\frac{3\beta }{16\alpha \sqrt{1-\zeta^{2}}}A_{0}{}^{2}\left(1-e^{-2\zeta \omega_{n}t}\right)\right]$$

According to the time-domain attenuation curve of the free vibration of the system and the Hilbert signal processing method, the system envelope curve is obtained and its backbone curve is drawn. The least-squares method is used to linearly fit the backbone curve to determine the system stiffness coefficient, and then the system damping coefficient is identified by the logarithmic attenuation method.

For identification of system stiffness coefficient, according to Equation (8), we draw the backbone curve of the system and fit the backbone curve to a straight line by the least-squares method, which is recorded as

(12) $$y\approx b_{0}x+b_{1}$$

Then, we combine Equations (8) and (12) and simplify the combination to obtain

(13) $$\alpha \approx b_{1}m/\left(1-\zeta^{2}\right)$$

(14) $$\beta \approx 4b_{0}m/3$$

For identification of system damping coefficient, for the PEH-C, the envelope under free vibration attenuation can be fitted with an exponential function, and its equation is set as

(15) $$A\left(t\right)\approx Ae^{at}$$

Combining Equations (6), (11) and (15), we can obtain

(16) $$\zeta \approx -a/\sqrt{\alpha /m\;}$$

(17) $$c=2\zeta \sqrt{\alpha m}\approx -2ma$$

Combining Equations (13) and (16), we can obtain

(18) $$\alpha \approx \frac{b_{1}m}{1-ma^{2}/\alpha }$$

Equation (18) can be solved in parallel with vertical Equations (16) and (17) to obtain the linear stiffness coefficient α, damping ratio coefficient ζ, and damping coefficient c of the system:

(19) $$\alpha \approx b_{1}m+ma^{2}$$

(20) $$\zeta \approx -a/\sqrt{b_{1}+a^{2}}$$

(21) $$c=2\zeta \sqrt{\alpha m}=-2ma$$

For the identification of the system electromechanical coupling coefficient and equivalent capacitance coefficient of the system, we make the system response output of the electrical equation in the dynamic equation in an open-circuit state, take $R=10^{7}\Omega$, and add a sinusoidal excitation to make the system output stable at a fixed frequency. At this time, the output voltage, voltage change rate, and velocity signal response output can be regarded as the first-order harmonic state, namely

(22) $$\dot{r_{1}}\left(t\right)=p_{0}\sin\left(\omega t\right)+q_{0}\cos\left(\omega t\right)\;$$

(23) $$\dot{v}\left(t\right)=p_{1}\sin\left(\omega t\right)+q_{1}\cos\left(\omega t\right)$$

(24) $$v\left(t\right)=p_{2}\sin\left(\omega t\right)+q_{2}\cos\left(\omega t\right)$$

In the equation, $p_{0}$, $q_{0}$, $\;p_{1}$, $\;q_{1}$, $\;p_{2}$ and $q_{2}$ are coefficients, which are measured by experiment. Substituting Equations (22)–(24) into Equation (3) and solving them, we can obtain

(25) $$\vartheta \left[p_{0}\sin\left(\omega t\right)+q_{0}\cos\left(\omega t\right)\right]+c_{p}\left[p_{1}\sin\left(\omega t\right)+q_{1}\cos\left(\omega t\right)\right]+\frac{\left[p_{2}\sin\left(\omega t\right)+q_{2}\cos\left(\omega t\right)\right]}{R}=0\;$$

Since Equation (25) is constant, the coefficients of the sin term and cos term of the equation can be set to 0 at this time, and Equation (25) can be solved to obtain the system electromechanical coupling coefficient ϑ and the equivalent capacitance coefficient $c_{p}$ expression:

(26) $$\vartheta =\frac{p_{2}q_{1}-p_{1}q_{2}}{R\left(p_{1}q_{0}-p_{0}q_{1}\right)}$$

(27) $$c_{p}=\frac{q_{2}p_{0}-p_{2}q_{0}}{R\left(p_{1}q_{0}-p_{0}q_{1}\right)}$$

## 4. The Verification of Parameter Identification Value

### 4.1. Numerical Verification of Mechanical Parameter Identification

In order to verify the correctness of the system’s mechanical and electrical parameter modeling, the relevant parameters in the system dynamics equation are selected for numerical simulation. The parameter values are shown in Table 1.

**Figure 3:**
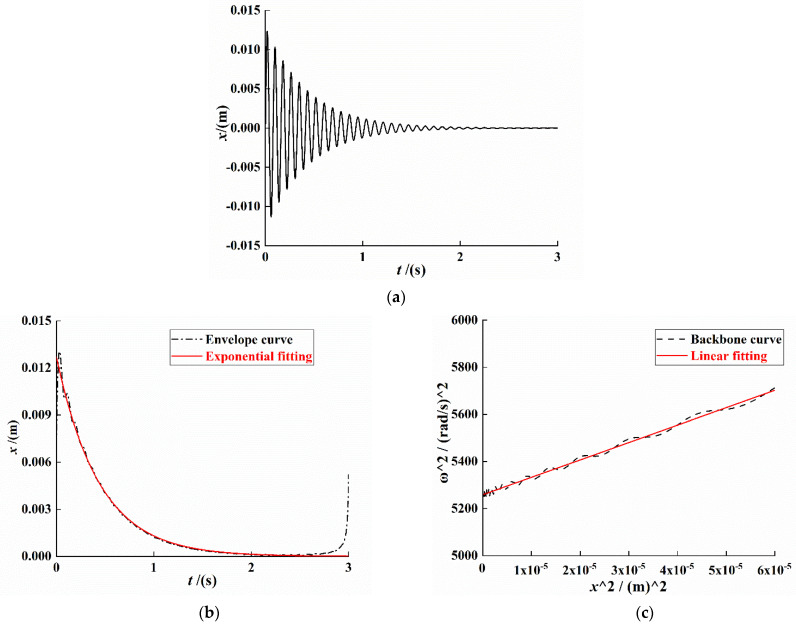
System response curve under free vibration attenuation. (**a**) Attenuated signal curve. (**b**) Signal envelope curve. (**c**) Signal backbone curve.

For the system of Equation (4), the unit pulse excitation δ(t) is selected for simulation analysis, and the Runge–Kutta algorithm is used to solve the dynamic system. Subsequently, the Hilbert transform method is used to process the time-domain attenuation signal; the system vibration attenuation curve, envelope, and backbone curve are drawn; and we use the least-squares method to fit the envelope and backbone curve data. The result is shown in Figure 3.

As shown in Figure 3b, the fitting equation of the envelope of the system displacement attenuation curve can be expressed as

(28) $$y\approx 0.1268e^{-2.27t}$$

As shown in Figure 3c, the system backbone curve fitting equation can be expressed as

(29) $$y\approx 7.391\times 10^{6}t+5259.042$$

From Equations (28) and (29), a=−2.27, $b_{0}=7.391\times 10^{6}$, and $b_{1}=5259.042$. Substituting the values of $a,b_{0}$, and $b_{1}$ into Equations (19), (14) and (21), the system linear stiffness coefficient α, nonlinear stiffness coefficient β, and linear damping coefficient c can be obtained: α≈22.11, β≈41,389.6, and c≈0.0191.

In order to analyze the accuracy of the identification algorithm, an error analysis of the identification coefficient is performed, as shown in Table 2.

It can be seen from Table 2 that the identification error of the damping coefficient based on the logarithmic attenuation method is only 4.5%. According to the backbone curve of the free attenuation signal, the errors of the linear stiffness and the nonlinear stiffness are obtained by the identification; they are 0.43% and 1.37%, respectively. The errors are less than 5%, and the errors of the last two are even less than 2%, which is within an acceptable range. Therefore, the identification method has good accuracy.

### 4.2. Numerical Verification of Electrical Parameter Identification

**Figure 4:**
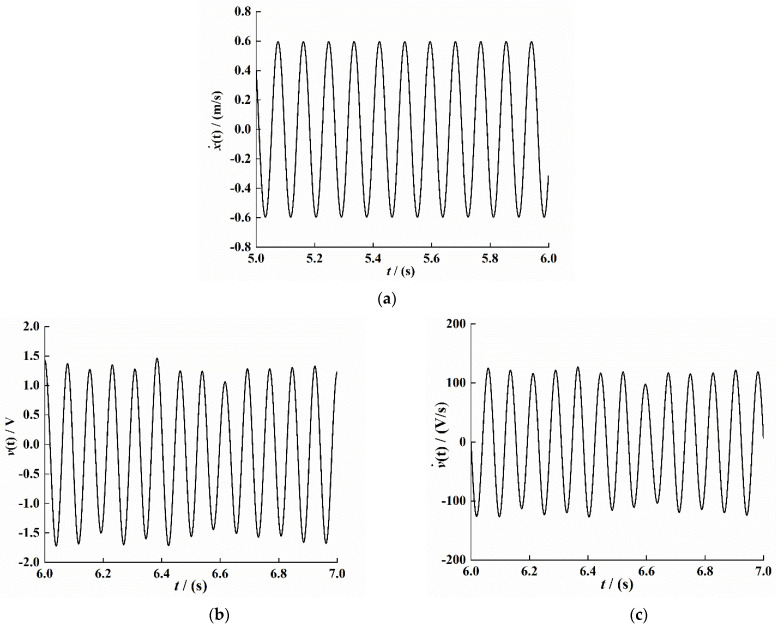
Response characteristic curve under system stability (*t* = 5–6 s). (**a**) Velocity signal. (**b**) Voltage signal. (**c**) Voltage change ratio signal.

According to the above-given conditions, Equation (3) is dynamically solved and analyzed by the Runge–Kutta numerical method, and the stable time period data are selected to obtain the system response characteristic curve. The simulation result is shown in Figure 4.

The least-squares method is used to curve-fit the time–velocity signal, time–voltage signal, and time–voltage change ratio signal in Figure 4; the fitting results are as follows:

(30) $$\dot{r_{1}}\left(t\right)=-0.2084\times \sin\left(72.5\times t\right)-0.5671\cos\left(72.5\times t\right)$$

(31) v(t)=20.08×sin(72.5×t)−8.056×cos(72.5×t)

(32) $$\dot{v}\left(t\right)=530.9\times \sin\left(72.5\times t\right)+1476\times \cos\left(72.5\times t\right)$$

According to Equations (30)–(32),$\;p_{0}=-0.2084$, $p_{1}=530.9$, $p_{2}=20.08$, $q_{0}=-0.5671$, $q_{1}=1476.3$, and $q_{2}=-8.06$. Substituting these values into the Equations (26) and (27), the system electromechanical coupling coefficient ϑ and the equivalent capacitance coefficient $c_{p}$ value can be obtained:

(33) $$\vartheta =5.1977\times 10^{-4}$$

(34) $$c_{p}=2.0025\times 10^{-7}$$

In order to analyze the accuracy of the identification algorithm, the electromechanical coupling coefficient ϑ and the equivalent capacitance coefficient $c_{p}$ are obtained by the identification and analyzed for error, and the results are shown in Table 3.

It can be seen from Table 3 that the errors of the electromechanical coupling coefficient and equivalent capacitance coefficient of the system are 0.11% and 0.13%, respectively, which is mainly due to a certain error in the first harmonic fitting of the system response signal. The error of both is less than 0.5%; therefore, the identification method has good accuracy.

## 5. Experimental Analysis

**Figure 5:**
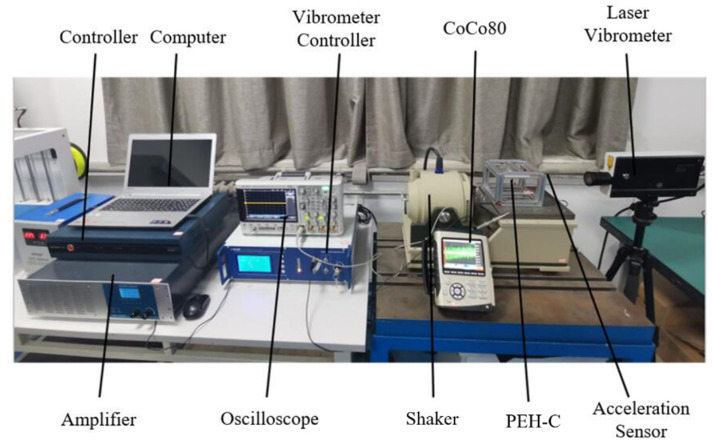
Experimental platform for vibration test of the PEH-C.

**Figure 6:**
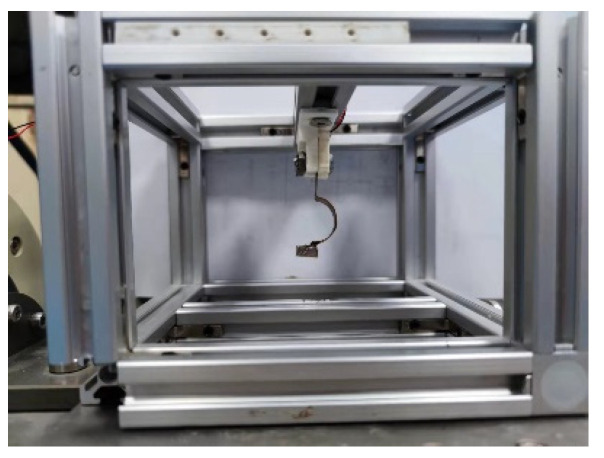
PEH-C.

In order to obtain the actual dynamic model parameter values of the piezoelectric energy harvester and verify the correctness of the parameter identification method, an experimental platform was built for experimental verification, as shown in Figure 5 and Figure 6. The experimental platform is composed of laser vibrometer (LV-S01), vibrometer controller (LV-S01), oscilloscope (DSOX3024T), shaker (E-JZK-5T), handheld vibrometer (CoCo-80), computer (Lenovo), controller (VT-9008), amplifier (E5871A) and acceleration sensor; the resolution of laser vibrometer was 1 μm/s. During the experiment, we used the laser vibrometer to collect the response output velocity signal of the PEH-C and used the handheld vibrometer for data collection and storage; next, under the excitation conditions, the output terminal of PEH-C was directly connected to the oscilloscope probe, and the voltage signal was obtained through the oscilloscope. The length of the composite beam in the *X*-axis direction was 40 mm, the width of the composite beam was 8 mm, the thickness was 0.2 mm, the length of the linear beam was 20 mm, and the radius and chord length of the arched part were 10 mm and 20 mm. The width of the PVDF pasted on the composite beam was the same as that of the composite beam; the thickness was 0.11 mm.

**Figure 7:**
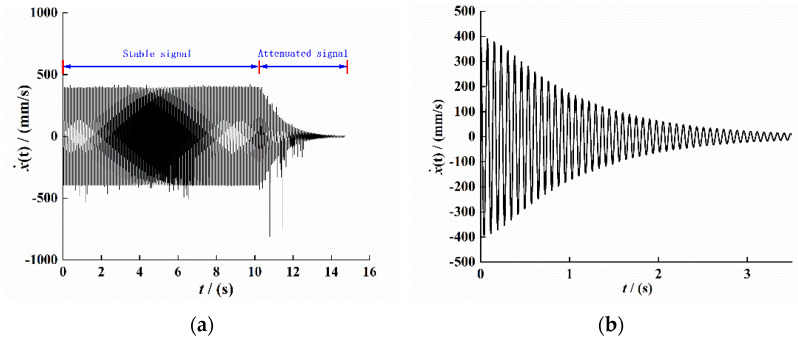

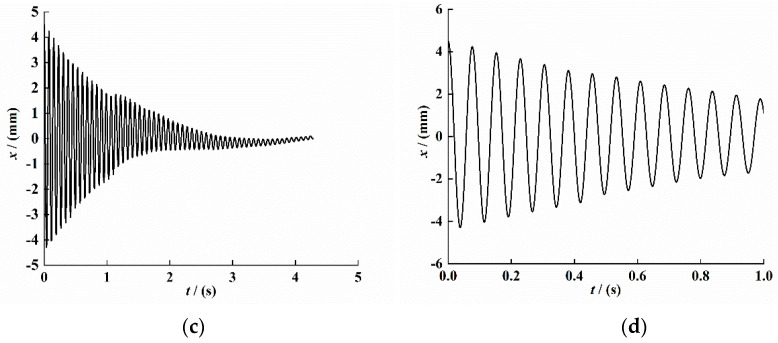
The response to time-domain signals of the PEH-C. (**a**) Original Velocity signal. (**b**) Velocity signal after VMD filtering. (**c**) Displacement attenuation signal. (**d**) Displacement attenuation sinal.

We set the excitation amplitude of the vibration platform to $10\;m/s^{2}$ and the excitation frequency to 13 Hz, and we conducted a fixed frequency experiment under simple harmonic excitation. The result is shown in Figure 7. Figure 7a shows the original velocity data signal collected by CoCo80. The signal has an obvious external interference signal, which is mainly caused by the external environment. We intercepted the signal of the attenuation part and conducted variational mode (VMD) processing to filter out the interference signal, and we obtained the velocity signal under the open-circuit condition of the composite beam piezoelectric vibrator, as shown in Figure 7b. As shown in Figure 7c, the displacement signal was obtained by integrating the velocity signal. The image has obvious distortion in 2–4 s, indicating that the envelope of the displacement signal is no longer smooth at this time, which may be caused by the VMD decomposition filtering of the signal and the signal curve not being smooth. We intercepted the 1-s signal, and the result is shown in Figure 7d.

**Figure 8:**
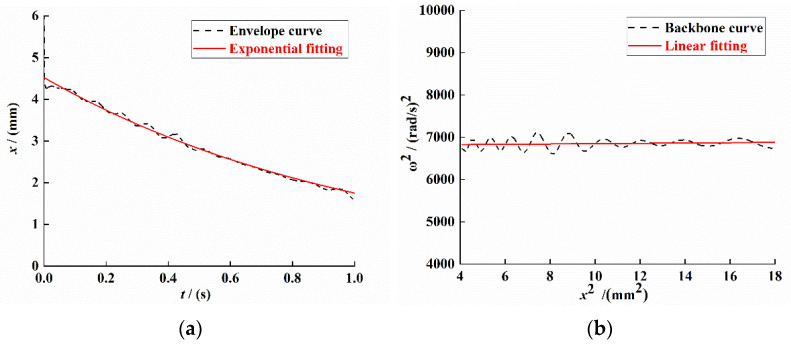
System response curve under free vibration attenuation. (**a**) Signal envelope curve. (**b**) Signal backbone curve.

For the signal data shown in Figure 7d, the Hilbert transform method was used to process the displacement time-domain attenuation signal and draw its envelope and backbone curve; the results are shown in Figure 8a,b.

It can be seen from Figure 8a that the expression of the fitting equation for the envelope of the displacement attenuation curve is

(35) $$y\approx 4.5244e^{-0.9484t}$$

It can be seen from Figure 8b that the expression of the system backbone curve fitting equation is

(36) $$y\approx 3.809\times 10^{6}t+6812.33$$

From Equations (35) and (36), a=−0.9484, $b_{0}=3.809$, and $b_{1}=6812.33$. Substituting the values of a, $b_{0}$, and $b_{1}$ into Equations (19), (14) and (21), the actual linear stiffness coefficient α, the nonlinear stiffness coefficient β, and the linear damping coefficient c in the dynamic model of the PEH-C can be obtained:

(37) $$\alpha \approx b_{1}m+ma^{2}=4.2\times 10^{-3}\times \left(6812.33+{\left(-0.9484\right)}^{2}\right)=28.62$$

(38) $$\beta \approx \frac{4b_{0}m}{3}=\frac{4\times 3.809\times 4.2\times 10^{3}}{3}=21330.6$$

(39) $$c\approx -2ma=2\times 4.2\times 10^{-3}\times 0.9484=0.008$$

**Figure 9:**
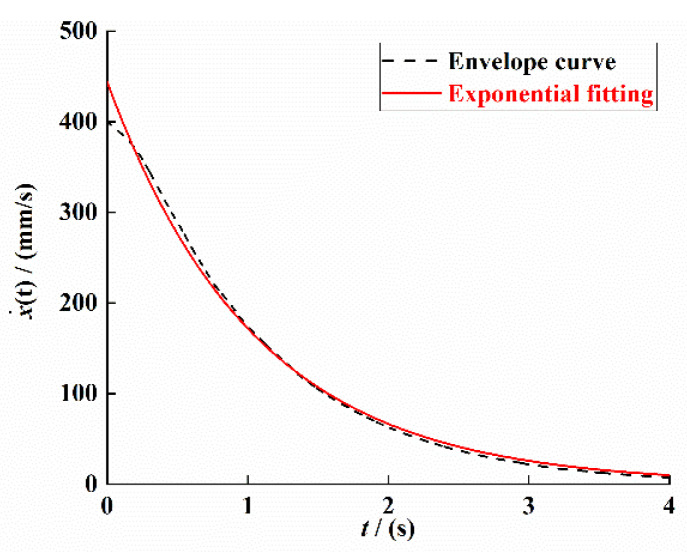
Velocity attenuation signal envelope and its fitting curve.

In order to ensure the accuracy of the damping coefficient of the PEH-C, the velocity attenuation signal in Figure 8b was processed by Hilbert transform to obtain its envelope; finally, the curve fitting was performed based on the least-squares method. The result is shown in Figure 9.

The envelope curve fitting equation is

(40) $$y\approx 443.8e^{-0.9483t}$$

Thus, we can obtain the size of the damping coefficient as 0.008; the comparison shows that the damping coefficient identified based on the velocity attenuation curve is consistent with the identification result based on the displacement attenuation signal.

**Figure 10:**
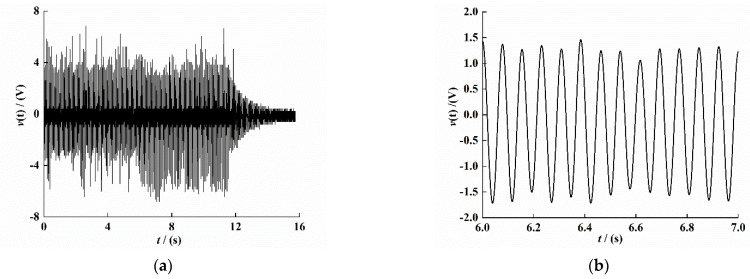

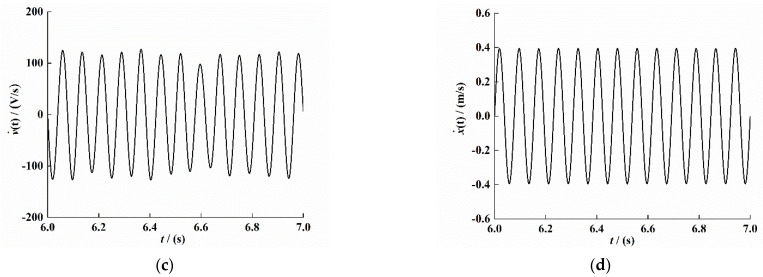
Response time-domain curve of the PEH-C. (**a**) Original voltage signal. (**b**) Voltage signal (*t* = 6–7 s). (**c**) Voltage change rate signal (*t* = 6–7 s). (**d**) Velocity signal (*t* = 6–7 s).

Figure 10a shows the output voltage diagram of the PEH-C under the same conditions. In order to eliminate the interference of hybrid wave on the signal, the experimental data were filtered, and the steady-state output voltage was intercepted within 6–7 s. The result is shown in Figure 10b; the time interval in which the voltage is negative at 6–8 s is significantly longer than the time interval in which the voltage is positive. The voltage signal in the figure is obviously asymmetric, and the voltage is not completely stable, which may be caused by the manufacturing error of the PEH-C and the arched section of the composite beam. Figure 10c shows the voltage change rate curve at the same time. In order to avoid the problem of asynchrony when collecting voltage and velocity signals, according to the theoretical simulation results, the velocity signal change trend should be 180° out of phase with the voltage change rate trend. The result is shown in Figure 10d.

As shown in Figure 10, we used the least-squares method to curve-fit the time–voltage signal (Figure 10b), time–voltage change rate signal (Figure 10c), and time–velocity signal (Figure 10d), The fitting results are as follows:

(41) v(t)=−0.135×sin(81.68×t)+1.44×cos(81.68×t)

(42) $$\dot{v}\left(t\right)=-118\times \sin\left(81.68\times t\right)-5.86\times \cos\left(81.68\times t\right)$$

(43) $$\dot{r_{1}}\left(t\right)=0.394\times \sin\left(81.68\times t\right)+0.00265\times \cos\left(81.68\times t\right)$$

From Equations (41)–(43), we can see that $p_{0}=0.394$, $p_{1}=-118$, $p_{2}=-0.135$, $q_{0}=0.00265$, $q_{1}=-5.86$, and $q_{2}=1.44$. Substituting these values into Equations (26) and (27), the system electromechanical coupling coefficient ϑ and the equivalent capacitance coefficient $c_{p}$ value can be obtained:

(44) $$\vartheta =8.57\times 10^{-5}$$

(45) $$c_{p}=2.85\times 10^{-7}$$

According to Equation (41), the system resonance frequency $\omega_{n}=81.68\;\mathrm{rad}/s$ and the simultaneous equation $\omega_{n}=\sqrt{\alpha /m}$; we bring the mechanical parameter α into it, and therefore, α=28.02, Table 4 shows the identification results of system mechanics and electrical parameters.

**Figure 11:**
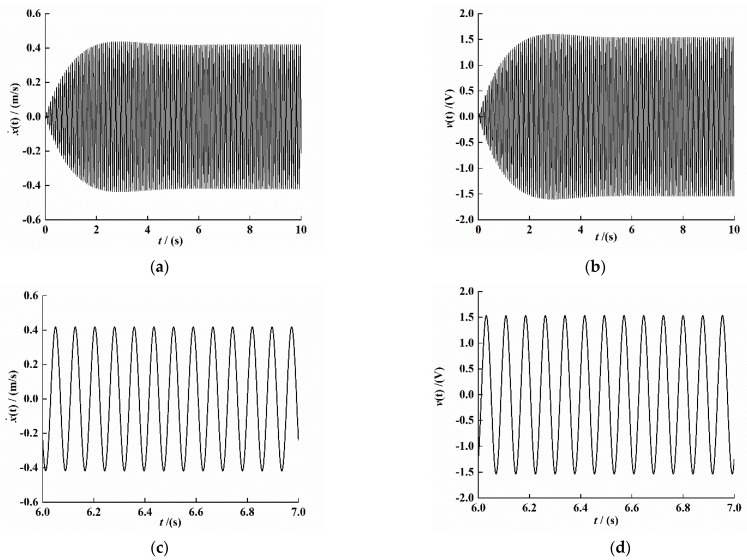
System numerical simulation response time-domain signal. (**a**) Velocity signal. (**b**) Voltage signal. (**c**) Velocity interception signal under stability. (**d**) Voltage interception signal under stability.

We substitute the identification result into (3) for a solution and give the system external excitation as sin(81.68×t); the simulation result is shown in Figure 11.

Comparing Figure 7, Figure 10 and Figure 11, it can be seen that the theoretical analysis is consistent with the experimental results, and it is not difficult to find that the identification method has good accuracy.

## 6. Conclusions

Based on the unique short-circuit and open-circuit output characteristics of the PEH-C, this paper proposes a high-precision identification method for the damping coefficient, stiffness coefficient, electromechanical coupling coefficient, and equivalent capacitance coefficient of the PEH-C. The analytical expressions of the parameters are obtained through theoretical modeling, and then the Runge–Kutta numerical method is used to verify the accuracy of the algorithm. The results show that the error of the damping coefficient identified by the algorithm is 4.5%; the errors of linear stiffness and nonlinear stiffness are 0.43% and 1.37%, respectively; and the errors of electromechanical coupling coefficient and equivalent capacitance coefficient are 0.11% and 0.13%, respectively. An experimental platform was built for verification, and the experimental results show that the parameters obtained through parameter identification have good accuracy and applicability. The error in this study is less than 5%, which is within an acceptable range when compared with the error analysis of related studies in the reference literature. We can use this parameter identification method to obtain the mechanical and electrical parameters of the system, solve the system dynamics model more accurately, and better guide the design of piezoelectric energy harvesters.

## Figures and Tables

**Table 1:** The parameter value table of the energy capture system.

Parameter	Name	Value	Unit
m	composite beam mass	0.0042	Kg
c	the first-order damping of composite beam	0.02	Ns/m
α	linear stiffness coefficient	22.1	N/m
β	nonlinear stiffness coefficient	41,963	N/m^3^
$H_{s}$	basic excitation coefficient	0.004016	Kg
$c_{p}$	equivalent capacitance coefficient of piezoelectric film	2 × 10^−7^	C
ϑ	electromechanical coupling coefficient	5.192 × 10^−4^	C/m
R	load resistance	1 × 10^7^	
$\ddot{z}\left(t\right)$	vibration acceleration	8×sin(72.5t)	$m/s^{2}$

**Table 2:** Identification results of mechanical parameters.

Parameter Type	*c*	α	β
Select value	0.02	22.1	41,963
Identification value	0.0191	22.1096	41,389.6
Error	4.5%	0.43%	1.37%

**Table 3:** Results of electrical parameter identification.

Parameter Type	ϑ	$c_{p}$
Select value	$5.192\times 10^{-4}$	$2\times 10^{-7}$
Identification value	$5.1977\times 10^{-4}$	$2.0025\times 10^{-7}$
Error	0.11%	0.13%

**Table 4:** The identification results of the mechanical and electrical parameters of the PEH-C.

Parameter	α	β	c	ϑ	$c_{p}$
Identification value	28.02	21,330.6	0.008	$8.57\times 10^{-5}$	$2.85\times 10^{-7}$

## Data Availability

Not applicable.
